# Complex axial growth patterns in an early Cambrian trilobite from South Australia

**DOI:** 10.1098/rspb.2021.2131

**Published:** 2021-12-22

**Authors:** James D. Holmes, John R. Paterson, Diego C. García-Bellido

**Affiliations:** ^1^ Department of Earth Sciences, Palaeobiology, Uppsala University, Villavägen 16, Uppsala 752 36, Sweden; ^2^ School of Biological Sciences, University of Adelaide, North Terrace, Adelaide, South Australia 5005, Australia; ^3^ Palaeoscience Research Centre, School of Environmental and Rural Science, University of New England, Armidale, New South Wales 2351, Australia; ^4^ South Australian Museum, North Terrace, Adelaide, South Australia 5000, Australia

**Keywords:** arthropod, sexual maturity, allometry, evo-devo, ontogeny, morphometrics

## Abstract

The exceptional fossil record of trilobites provides our best window on developmental processes in early euarthropods, but data on growth dynamics are limited. Here, we analyse post-embryonic axial growth in the Cambrian trilobite *Estaingia bilobata* from the Emu Bay Shale, South Australia. Using threshold models, we show that abrupt changes in growth trajectories of different body sections occurred in two phases, closely associated with the anamorphic/epimorphic and meraspid/holaspid transitions. These changes are similar to the progression to sexual maturity seen in certain extant euarthropods and suggest that the onset of maturity coincided with the commencement of the holaspid period. We also conduct hypothesis testing to reveal the likely controls of observed axial growth gradients and suggest that size may better explain growth patterns than moult stage. The two phases of allometric change in *E. bilobata*, as well as probable differing growth regulation in the earliest post-embryonic stages, suggest that observed body segmentation patterns in this trilobite were the result of a complex series of changing growth controls that characterized different ontogenetic intervals. This indicates that trilobite development is more complex than previously thought, even in early members of the clade.

## 1. Introduction

Trilobites are some of the most abundant early animal fossils with a record spanning almost the entire Palaeozoic and are useful for answering questions about early animal evolution, including those relating to developmental processes (e.g. [1–3]). Unlike most fossil groups, development in trilobites is well known due to their possession of a biomineralized exoskeleton throughout the majority of post-embryonic ontogeny [4,5]. Many articulated trilobite ontogenies have been published, particularly in recent years (e.g. [6–9]). However, even with their exemplary record, obtaining the data required for detailed morphometric studies, such as those relating to segmental growth, is problematic. Thus far, detailed studies modelling trilobite development are limited to three species: *Aulacopleura koninckii* from the Silurian of the Czech Republic (for a review see [1]); *Elrathia kingii* from the Cambrian (Miaolingian) of Utah, USA [9] and *Oryctocarella duyunensis* from the Cambrian Series 2 of Hunan Province, China [10]. In these species, axial growth gradients have been identified in the trunk, showing higher rates of growth at the posterior and lower rates at the anterior (and with opposite polarity in the cephalon of *A. koninckii* [11]). These studies have generally used growth stages associated with the moult cycle as an explanatory variable of trunk segment size patterns. This is in part due to the general assumption that growth of external structures in euarthropods is often characterized by a constant per-moult growth rate, the so-called Dyar's rule [12]. It has been suggested that trilobite growth largely conforms to Dyar's rule [2].

Trilobite post-embryonic ontogeny followed a stepwise progression due to the moult cycle and has generally been divided into three major periods. During the initial protaspid period, the dorsal exoskeleton was composed of a single, fused plate. The meraspid period commenced when an articulation formed between the cephalon and the trunk. During the meraspid period, segments were generally added at a subterminal generative zone near the posterior of the pygidium and released from the anterior to become fully articulating segments of the thorax—rates and timing of segment production and release vary between taxa [5]. The holaspid period commenced when the full number of thoracic segments was achieved. Trilobites generally display what is termed hemianamorphic development, with an increasing number of segments during an initial anamorphic phase, followed by an epimorphic phase with continued moulting and growth after a stable segment number had been reached [5]. This growth mode occurs across several extant euarthropod groups, such as myriapods (including in both diplopods and chilopods), pancrustaceans (e.g. branchiopods, copepods and decapods) and pycnogonids [13]. Based on the phylogenetic distribution of this and other growth modes across the euarthropod tree, hemianamorphosis likely represents the ancestral condition for the phylum [5].

Newly acquired material from the Cambrian Series 2 (Stage 4) Emu Bay Shale of South Australia includes very large numbers of the ellipsocephaloid trilobite *Estaingia bilobata*. We recently presented data [6] on 124 *E. bilobata* meraspides for which the degree (a morphotype with the same number of thoracic segments, see [14]) could be identified with a high level of confidence. Each meraspid degree was interpreted as a separate developmental stage or moult instar (except the first ‘M0’ meraspid degree with no thoracic segments that may have had multiple stages). In *E. bilobata*, as in many trilobites, a segment was released from the anterior of the pygidium into the thorax at each moult during the meraspid period, such that successive stages are represented by instars with a steadily increasing number of thoracic segments. The holaspid period commenced when 13 thoracic segments were attained. *Estaingia bilobata* displays protomeric (specifically hypoprotomeric) growth [5], with the epimorphic phase commencing prior to the holaspid period (at stage D10, with 10 thoracic segments). Here, we explore patterns of axial growth across the post-embryonic ontogeny of *E. bilobata*, including the testing of different growth gradient hypotheses, to reveal likely growth control mechanisms in this early euarthropod.

## 2. Material and methods

### (a) Specimen data

Material considered in this study was collected between 2007 and 2019 from the Emu Bay Shale at Big Gully on the north coast of Kangaroo Island [15] and is housed in the South Australian Museum Palaeontological collections. There are approximately 650 registered specimens of *E. bilobata* in the collection, with many additional unregistered specimens associated with other fossils. The analyses conducted here are based on 99 meraspides and 135 holaspides (total *n* = 234) for which accurate axial length measurements could be taken (full dataset provided in the electronic supplementary material). Not all measures could be taken from each specimen, so various analyses are based on subsets of this dataset. The meraspid segmental growth gradient analysis was conducted on 97 specimens from stages D1 to D12 (with 1–12 thoracic segments: 7 D1, 3 D2, 8 D3, 7 D4, 4 D5, 8 D6, 16 D7, 11 D8, 7 D9, 6 D10, 14 D11, 6 D12). Two additional ‘M0’ meraspides (with no thoracic segments), both considered to be of the preceding stage to D1, are included in other analyses where appropriate (as stage D0).

### (b) Measurements

Methodology for the collection of body part length data largely follows Fusco *et al.* [16]. Meraspides and small holaspides were photographed using an Olympus SZX7 stereomicroscope with an Olympus SC50 camera attachment and the associated Olympus cellSens Standard v. 1.17 software. Larger specimens were photographed with a Canon EOS 50D Digital SLR camera and the Canon EOS Utility 2.8.1.0 program, either with a Canon EF-S 60 mm 1 : 2.8 macro lens or an MP-E 65 mm 1 : 2.8 1–5× macro lens. Using the freeware vector-based drawing program Inkscape (v. 0.92), a sagittal line along the entire body was drawn on each specimen, and lateral lines were drawn between points on either pleural lobe where each articulation bends sharply at the fulcrum. In *E. bilobata*, this corresponds to the most distal point of the inner, straight portion of each articulation (electronic supplementary material, figure S1). These modified images were imported into ImageJ [17], and a series of landmarks placed along the sagittal line at the intersections with the anterior cephalic margin, glabellar anterior, occipital furrow, posterior pygidial margin and one for each of the line intersections representing the articulations; images were calibrated using the scale bar. These data were imported into the R statistical environment and manipulated to produce a dataset containing the lengths of all body parts at each stage. In our analyses, the boundary between cephalon and thorax corresponds to the intersection of the line drawn across the anterior of the first thoracic segment (TS1) and the sagittal line and is slightly anterior of the true posteriormost axial point of the cephalon. This also means that the occipital ring length (ORL) measure excludes the posterior part of the occipital ring. Mean length was calculated for each body part at each stage, as well as mean relative thoracic segment length (RLS) and mean relative position of the posterior boundary of each thoracic segment (RPS) relative to trunk length (TRL). See the electronic supplementary material for details on how these measures were calculated. All data analysis and basic figure production were conducted in R, with R scripts provided in the electronic supplementary material. Figures were refined in Inkscape (v. 0.92).

### (c) Axial allometric analyses

Relationships between body section lengths with respect to more inclusive regions were examined across ontogeny, i.e. cephalic length (CEL) and TRL with respect to body length (BOL), thoracic segment length (LTS) and pygidial length (PYL) with respect to TRL, and frontal area length (FAL), pre-occipital glabellar length (PGL) and ORL with respect to CEL. Preliminary data exploration revealed that these relationships are well explained by the standard allometric model of a constant ratio between differential growth rates (i.e. log–log linear relationships). However, many body sections underwent seemingly abrupt changes in trajectories close to the anamorphic/epimorphic and meraspid/holaspid transitions. Subsequently, we fitted two-phase (one threshold) segmented linear regression models to these log–log relationships using the function *chngptm()* from the R package *chngpt* [18]. This function fits an optimal threshold model using maximum likelihood and provides an estimate of the associated threshold or ‘change point’—the point of ontogeny where the abrupt change in slope (or allometric coefficient) occurs. Change point confidence intervals were estimated with the recommended bootstrap method (1000 replicates).

### (d) Initial growth gradient detection

**Figure 1.**
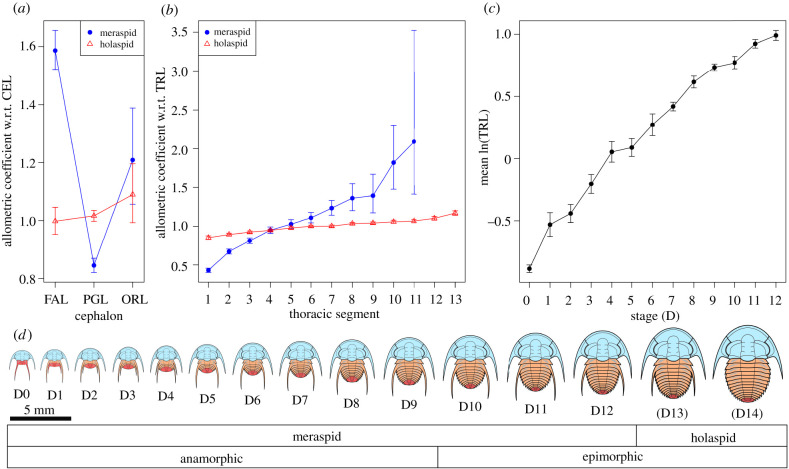
Allometric coefficients of cephalic axial lengths (*a*) and thoracic segments (*b*) during the meraspid and holaspid periods of *Estaingia bilobata*. There is a clear gradient in the thorax, with higher rates of growth towards the posterior, becoming flatter in the holaspid period. Cephalic growth is more complex although a high growth rate at the anterior is evident during the meraspid period. Note that meraspid thoracic segment 12 is omitted from (*b*) as the estimate is very high [4.28] with wide confidence intervals [3.09, 6.77]. (*c*) Mean trunk length showing a decreasing growth rate across the meraspid period. (*d*) Ontogenetic series of *E. bilobata* showing the meraspid and earliest holaspid periods. CEL, cephalic length; TRL, trunk length; FAL, frontal area length; PGL, pre-occipital glabellar length; ORL, occipital ring length; w.r.t., ‘with respect to’. Bars are 95% CIs. (Online version in colour.)

Previous studies have used both the average growth rate (AGR, see [2]) of thoracic segments across ontogeny to reveal trunk growth gradients, as well as the allometric coefficients of thoracic segments with respect to TRL (using mean values at each stage) [7,16]. In this case, AGR is an inappropriate measure due to the clear decrease in the growth rate of the various thoracic segments across meraspid ontogeny (electronic supplementary material, figure S2*a*). As such, we use allometric coefficients of thoracic segments to illustrate growth gradients in the trunk of *E. bilobata*. Major axis regression was conducted on the log-transformed lengths of each thoracic segment against TRL for both the meraspid and holaspid periods. Unlike the stage data, the relationship between log length of the various thoracic segments and trunk was well explained by a linear relationship. The slopes of these regressions represent the allometric coefficients for each thoracic segment with respect to TRL. These show a clear growth gradient in the trunk, with anterior segments displaying lower allometric coefficients with respect to TRL and more posterior segments showing higher values (figure 1*b*). Growth in the cephalon was more complex (figure 1*a*, see below).

### (e) Growth gradient hypothesis testing

Fusco *et al*. [11,16] tested two models of segmental growth in the Silurian trilobite *Aulacopleura koninckii*: the segmental gradient (SG) and trunk gradient (TG) hypotheses. The SG hypothesis expects a constant growth rate of individual segments once they are released into the thorax, with overall trunk growth dependent upon the autonomous growth of individual thoracic segments (in addition to the pygidium). This reflects the standard model of allometric growth, where the relationship in size between two body measurements is the result of differential constant growth rates [19]. By contrast, the TG hypothesis predicts a decrease in segment growth rates across ontogeny, due to segments shifting their position from a posterior to a more anterior position in the trunk as segments are added at the posterior. Segments are thus sequentially exposed to decreasing values of the gradient. The models of Fusco *et al*. [11,16] incorporated a fixed trunk growth rate (TRG) based on their observations across the latter half of the meraspid period for *A. koninckii*. These models were adapted by Dai *et al*. [10] to test the SG and TG hypotheses in *Oryctocarella duyunensis*, after a general decrease in TRG was observed across the meraspid period. *Estaingia bilobata* shows decreasing growth rates across meraspid ontogeny for TRL, CEL and thoracic segments with respect to stage, *contra* Dyar's rule (figure 1*c*; electronic supplementary material, figure S2*a*,*b*). As such, the models used here to test the SG and TG hypotheses are the same as those of Dai *et al*. [10], and we include a modified TG model that uses stage instead of TRG for comparison (see below).

The SG hypothesis under the condition of a decreasing TRG suggests that each segment grew at some pre-defined rate proportional to the overall, changing TRG. Under this hypothesis, we tested two models: (i) the SG-R model, which sets the ratio of segment to trunk growth rates as constant; and (ii) the SG-A model, which sets a constant allometric coefficient for the segments with respect to the trunk (i.e. a constant ratio of logged segment/trunk growth rates). Both SG models have three parameters and are fitted using relative thoracic segment lengths (RLS). The TG hypothesis suggests that each segment grew at a rate specified by its position in the trunk at any one time; however, the condition of a changing TRG suggests that the gradient changed across ontogeny. We also tested two models under this hypothesis: (i) the TG-T model, which uses TRG as an explanatory variable and (ii) the TG-D model, which uses stage. The TG models do not rely on segments as individual units; rather, they recognize segmental boundaries as landmark positions within a continuous growth field. As such, they are best fitted using the relative position of posterior segmental boundaries (RPS). However, the model can be adapted to predict RLS, allowing direct comparison with the SG models. Thus, the TG-T_RLS_ and TG-D_RLS_ models use RLS, and the TG-T_RPS_ and TG-D_RPS_ models use RPS. Nonlinear least-squares regression was conducted in R with the *nlsLM()* function from the *minpack.lm* package [20], which uses a modification of the Levenberg–Marquardt algorithm. Model-fitting functions, procedures and R script files are provided in the electronic supplementary material.

## 3. Results

### (a) Axial allometric growth

**Figure 2.**
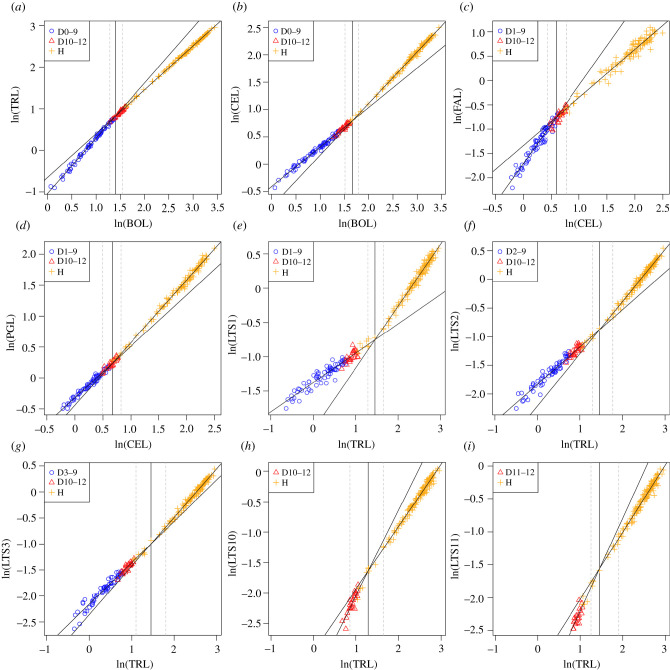
Threshold models fitted to various *Estaingia bilobata* body part axial lengths relative to a more inclusive body section. The solid vertical line in each plot represents the model change point estimate (the change in allometric coefficient, or slope), and the dashed lines represent 95% CIs. There are two phases of allometric changes: one in the late meraspid period involving changes in major body sections and the cephalon associated with the anamorphic/epimorphic transition (*a*–*d*), and the other in the early holaspid period involving changes in thoracic segments associated with the meraspid/holaspid transition (*e*–*i*). BOL, body length; LTS*i*, length of thoracic segment *i*; other abbreviations as per figure 1. (Online version in colour.)

Abrupt changes in growth trajectories occur in the axial lengths of a range of *E. bilobata* body sections. These are illustrated as log–log biplots of the various body sections relative to a more inclusive body region (figure 2). In general, these relationships are explained extremely well by the segmented linear threshold models, with changes occurring abruptly rather than gradationally. The change in slope of these models represents a change in allometric coefficient between the two variables in question at a specific point of ontogeny. An allometric coefficient of 1 represents isometric growth, whereas a coefficient greater or less than 1 represents positive or negative allometry, respectively. To allow for easy comparison, the change points of the models with CEL and TRL as explanatory variables were also standardized to BOL measurements based on threshold model estimates of BOL∼CEL and BOL∼TRL (table 1).

**Table 1.** Threshold model statistics discussed in the text. Models are described with the notation y ∼ x, where x represents the variable for which the threshold (change point) is being estimated. Measures in *italics* in the ‘BOL *estimated* change point’ column represent estimates based on threshold models of BOL∼CEL and BOL∼TRL. ‘Stage’ refers to the stages in which the change point estimate occurs within the size range for that stage (based on the threshold variable (x) of the original model). The ‘pre-AC’ and ‘post-AC’ columns are the slope estimates for the threshold models, representing the allometric coefficients before and after the estimated change points.

model	change point (mm)	BOL *estimated* change point (mm)	stage	pre-AC	post-AC
TRL∼BOL	BOL = 4.05	4.05	D10	1.33	1.05
CEL∼BOL	BOL = 5.29	5.29	H	0.72	0.94
FAL∼CEL	CEL = 1.81	*3.99*	D8–10	1.62	0.99
PGL∼CEL	CEL = 1.95	*4.42*	D11–12	0.83	1.01
LTS1∼TRL	TRL = 4.29	*7.37*	H	0.43	0.90
LTS2∼TRL	TRL = 4.29	*7.37*	H	0.65	0.91
LTS3∼TRL	TRL = 4.29	*7.37*	H	0.80	0.94
LTS10∼TRL	TRL = 3.63	*6.29*	H	1.40	1.02
LTS11∼TRL	TRL = 4.29	*7.37*	H	1.42	1.02

Changes in growth trajectories occur at two different stages of ontogeny. Firstly, the allometric coefficient of TRL relative to BOL drops in the late meraspid period (figure 2*a*), with a corresponding increase in that of CEL relative to BOL (figure 2*b*). An abrupt drop in the allometric coefficient of FAL relative to CEL occurs at the same time (figure 2*c*), as does a corresponding increase in that of PGL relative to CEL (figure 2*d*). As a whole, these changes occur in the late meraspid period, at or slightly after the anamorphic/epimorphic transition; CEL∼BOL (figure 2*b*) is the exception, suggesting a change very close to the meraspid/holaspid transition. The estimated change point of TRL∼BOL occurs at BOL = 4.05 mm (stage D10) and CEL∼BOL at BOL = 5.29 mm (the earliest holaspid period). The estimated change point of FAL∼CEL occurs a CEL = 1.81 mm (BOL = 3.99 mm; stages D8–10) and PGL∼CEL at CEL = 1.95 mm (BOL = 4.42; stages D11–12) (table 1).

By contrast, changes in the growth trajectories of thoracic segments occur in the early part of the holaspid period, after the meraspid/holaspid transition. The most anterior thoracic segments underwent a marked increase in allometric coefficient with respect to TRL (figure 2*e–g*), while towards the posterior, the reverse occurred (figure 2*h*,*i*). Intermediate segments show less change in the allometric coefficients, and as a result, threshold model change point estimates are uncertain, with wide confidence intervals (electronic supplementary material, table S1). Likewise, it is harder to detect change points in the most posterior segments, as these were only added late in meraspid ontogeny. Segments 1–3 have the highest number of data points, and the estimated change point for all three is TRL = 4.29 mm (BOL = 7.37 mm). Segments 10 and 11 are the only other segments with reasonable confidence intervals relative to the change point estimate. The estimated change point for segment 11 is again TRL = 4.29 mm, while segment 10 is TRL = 3.63 mm (BOL = 6.29 mm).

### (b) Growth gradient hypothesis testing

Based on comparison with the corrected Akaike information criterion (AIC_c_; table 2), it is clear that both the SG-A and TG-T_RLS_ models have much higher levels of support than the SG-R and TG-D_RLS_ models. Therefore, the possibility of a constant ratio of segment/trunk growth rates (SG-R model) is discounted, and it is clear that observed trunk growth rates between stages (TG-T_RLS_) are better at explaining the observed variation than stage (TG-D_RLS_). The SG-A (99.59%) and TG-T_RLS_ (99.56%) models explain a very similar amount of the observed variation in RLS. However, based on the AIC_c_ comparison, the SG-A model has a normalized probability *p* = 0.98 of being the correct model over the TG-T_RLS_ model with an evidential ratio (ER) of 53.07. The TG-T_RPS_ model (fitted using RPS) outperforms both the SG-A and TG-T_RLS_ models (fitted using RLS), explaining 99.82% of the observed variation. However, a direct comparison with AIC_c_ is not appropriate based on their differing response variables.

**Table 2.** Corrected Akaike information criterion (AIC_c_) comparison of the growth gradient models using relative thoracic segment length (RLS). The SG-A model is the best supported. AIC_c_, AIC_c_ score; ΔAIC_c_, difference in AIC_c_ score between the model in question and the model with the lowest score; wAIC_c_, probability of being the correct model among the set of competing models.

model	no. of par.	AICc	ΔAICc	wAICc
SG-A	3	−544.78	0.00	0.98
TG-T_RLS_	4	−536.84	7.94	0.02
TG-D_RLS_	4	−494.42	50.36	0.00
SG-R	3	−378.08	166.71	0.00

## 4. Discussion

### (a) Growth trajectory changes

The two phases of growth trajectory change recognized in *Estaingia bilobata* are likely associated with the two major ontogenetic transitions in this protomeric trilobite: the anamorphic/epimorphic and meraspid/holaspid transitions. The first phase involves broad changes in allometric coefficients of major body structures (CEL and TRL with respect to BOL) and of cephalic structures with respect to CEL. These occur at body sizes largely consistent with the late meraspid period. The second phase involves changes in segment growth rates with respect to TRL and occurs in the early holaspid period. In both phases, changes seem to occur soon after these transitions. This is most likely due to the timing of a change in growth control relative to the moult cycle. If a change in growth control occurred at the inception of the epimorphic phase (the transition from stages D9 to D10), a change in trajectory would not be observed until the transition from stages D10 to D11. Likewise, if a change in control occurred at the meraspid/holaspid transition, this would not manifest until the second moult stage of the holaspid period. Additional evidence in support of this comes from the loss of macropleural spines on the second thoracic segment at about the same time—this probably occurs at the transition from the first to second holaspid stages [6].

The magnitude of the abrupt change in allometric coefficients estimated by several of the threshold models is remarkable. In the thorax, the coefficient of LTS1 (length of thoracic segment 1) rose from 0.43 to 0.90, while that of LTS11 dropped from 1.42 to 1.02. Across the length of the thorax, the changes in allometric coefficients of segments with respect to TRL had the effect of an immediate, dramatic flattening of the growth gradient early in the holaspid period. Prior to this, growth rates were much lower and higher at the anterior and posterior of the thorax, respectively, allowing the more posterior segments to increase rapidly with respect to anterior segments. Thus, the flatter trunk gradient of the holaspid period essentially ‘locks in’ the pattern established by the steeper gradient of the meraspid period. The flatter gradient then allows a gradual evening of segment lengths across the remainder of ontogeny. Very large changes in allometric coefficients also occur in the cephalon. For example, the coefficient of FAL relative to CEL was initially 1.62, allowing rapid expansion of FAL (and corresponding retraction of the glabella) across the early part of the meraspid period. Again, this trend was terminated after the abrupt change near the anamorphic/epimorphic transition when the coefficient dropped to 0.99, essentially representing isometric growth for the remainder of ontogeny. The changes in allometric coefficients are effectively captured in figure 1*a*,*b*, although note that these are based on separate major axis regressions of meraspid and holaspid body-part lengths, rather than the threshold models that estimate the change points.

Studies using geometric morphometric landmark analysis to quantify trilobite shape (cranidial, cephalic or exoskeletal) have revealed similar patterns to those identified in *E. bilobata*, generally showing high early rates of allometric change decreasing across ontogeny (e.g. [9,21])—although allometries can persist even in later stages (e.g. [22,23]). However, as they quantify ‘overall’ morphologies, these shape analyses tend to show much higher amounts of variation between specimens than the traditional measures analysed here. This makes it difficult to determine if shape changes are abrupt or gradational, or to tease out more precise relationships between individual morphological variables. Despite this, it has been shown that threshold models are often better at explaining overall shape change across ontogeny than simple linear regression models and that, where known, these changes appear to occur close to the meraspid/holaspid transition (e.g. [21,24]). The interplay between overall shape and the more specific axial growth patterns identified here will be explored further in a future contribution using geometric morphometrics to quantify allometric shape change in *E. bilobata*.

### (b) Sexual maturity

In trilobites, the transition to the holaspid period has often been considered to coincide with the onset of sexual maturity, although no strong evidence has been presented to support such an assumption [5]. It has been suggested that such evidence may include changes in growth trajectories [25]. In extant euarthropods (e.g. crustaceans and myriapods), such changes often occur at important ontogenetic transitions, such as from larval to post-larval phases, anamorphic to epimorphic growth, or at sexual maturity [13]. Abrupt changes at (or close to) sexual maturity are particularly well-documented in extant brachyurids (crabs), with these generally manifesting as a sharp increase or decrease of allometric coefficients at the so-called puberty moult [26,27]. However, these changes are often associated with secondary sexual characters, e.g. increases in relative size in the male chelipeds and female abdomen. The changes observed in *E. bilobata* appear to be more closely associated with regulating overall body patterning and were apparently applicable to all individuals, with no observed sexual dimorphism in this taxon. Nevertheless, the observed changes—particularly those relating to segmental growth occurring well after the anamorphic/epimorphic transition—do suggest the transition to a mature phase.

The possible attainment of sexual maturity at (or prior to) the meraspid/holaspid transition rather than later in ontogeny is supported by the recent observation that the Cambrian (Stage 4) trilobite *Oryctocarella duyunensis* displayed determinate growth, with probably only one holaspid stage (the meraspid/holaspid transition most likely representing a terminal moult) [10]. By contrast, other trilobites (where known) displayed indeterminate growth, with continued growth and moulting throughout life. Interestingly, extant brachyurids show similar variation in this respect to trilobites, with indeterminate growth at one extreme and determinate growth (where the puberty moult is the terminal moult) at the other [27]. The meraspid/holaspid transition in *E. bilobata,* therefore, shows considerable similarity to the puberty moult of decapod crustaceans, occurring at a similar point in ontogeny and being associated with abrupt allometric changes.

The two phases of allometric change observed in the protomeric *E. bilobata* are also consistent with the decoupling of the anamorphic/epimorphic transition and sexual maturity in extant hemianamorphic myriapods, in which the latter is reached several moults after the former, as well as in euanamorphic species that never reach an epimorphic phase [13]. Furthermore, although morphometric studies on extant hemianamorphic euarthropods are rare, in the millipede *Glomeris balcanica*, growth trajectory changes may occur at both the anamorphic/epimorphic transition and subsequently at maturity [28]. However, growth in other trilobites can vary from the protomeric mode seen in *E. bilobata*. Examination of species where the anamorphic/epimorphic and meraspid/holaspid transitions occur coincidentally (the ‘synarthromeric’ mode), and where the latter precedes the former (‘protarthrous’) [5], will reveal how growth patterns across these transitions vary across Trilobita and help to refine comparisons with extant taxa.

### (c) Trunk growth patterns in *Estaingia bilobata*

In *E. bilobata*, the first thoracic segment (TS1) remains the longest throughout ontogeny, with segment lengths consistently decreasing towards the posterior. In *O. duyunensis*, TS2 is the longest for much of the meraspid period, shifting to TS3 at stage 11 [10]. In *Elrathia kingii*, TS2 is the longest in the early meraspid period, shifting to TS3–4 in later stages [9]. In *Changaspis elongata*, TS1–2 show similar lengths and are the longest in the early meraspid period, shifting to TS3 in later stages [7]. In the much younger *A. koninckii*, the longest segment in the early meraspid period is around TS4, shifting to TS6 at stage 17 and about TS9 in larger holaspides [11]. Interestingly, the trunk segment growth patterns identified across much of ontogeny for these trilobites only seem capable of producing a situation where TS1 is the longest thoracic segment in the earliest meraspid stages, in contrast to the observations outlined above. This suggests that these anteriormost segments were subject to different growth controls at the earliest stages of their development—probably within the protaspid and M0 pygidia—than segments that appeared later. This is the likely reason that a recently developed generative growth model [29] was only able to produce a trunk with posteriorly decreasing trunk segment lengths (using the example of *A. koninckii*). Furthermore, in *E. bilobata*, TS3–12 were approximately the same length when released from the pygidium, whereas TS1 and TS2 (particularly the former) were larger (electronic supplementary material, figure S3*a*). This may be linked to the presence of macropleural spines on both of these segments in early ontogenetic stages, which do not occur in the taxa discussed above. Another result of this is that the length of the released segment at each stage does not show an obvious proportional relationship to either the trunk or the pygidium. Rather, it shows an exponential decrease relative to TRL and a more complex relationship with PYL (electronic supplementary material, figure S3*b*,*c*). The length of the released segment in *A. koninckii* is apparently also complex, with no obvious relationship to stage, TRL or PYL (G. Fusco 2020, personal communication). It is possible that the release of segments is somehow connected to the maintenance of a constant absolute PYL across much of the meraspid period (see below).

For the majority of the anamorphic phase, the pygidium of *E. bilobata* had an extended equilibrium period where it contained six segments. Across this period (D0–10), the pygidial growth rate was essentially zero (non-significant ordinary least squares regression of PYL against stage, two-tailed Student's *t*-test, *n* = 99, *p* = 0.679; electronic supplementary material, figure S4). There is a decrease in pygidial size in stages D11–12 (and the earliest holaspid period) associated with onset of the epimorphic phase at D10 and the continued release of pygidial segments. The maintenance of a pygidium with a constant absolute length across the meraspid period is also seen in *A. koninckii* [11] and *E. kingii* [9], but not in *C. elongata* [7] or *O. duyunensis* [10].

### (d) Growth gradients: stage versus size?

The higher level of support for the SG hypothesis (based on models using RLS) reported here is at odds with the comparisons of Dai *et al*. [10] and Fusco *et al*. [11,16] that have generally endorsed the TG hypothesis. However, the TG-T_RPS_ model still explains the highest amount of observed thoracic segment variation in *E. bilobata*. The fact that TG-T (using TRG as an input variable) considerably outperforms TG-D (using stage) suggests that size may play a more important role than stage in determining relative thoracic segment lengths at different times. In general, *E. bilobata* body part size relationships are explained extremely well by the log–log segmented linear models employed here, whereas size∼stage relationships display complex decreasing growth rates over time (at odds with Dyar's rule). Furthermore, the two phases of allometric change in the late meraspid period and early holaspid period are likely to significantly affect any growth gradient analyses, in particular the change in TRL/CEL with respect to BOL in the late meraspid period. These allometries also render any estimates within the holaspid period made by model projections from the meraspid period questionable.

## 5. Conclusion

The identification of two phases of allometric change in *E. bilobata*, as well as inferred differing growth controls in the late protaspid/earliest meraspid period, suggest that observed body segmentation patterns in this trilobite were the result of a complex series of changing growth controls that characterized different intervals of post-embryonic ontogeny. Allometric changes associated with the meraspid/holaspid transition are suggested to signal the onset of sexual maturity. Results from similar studies on other trilobites, particularly those with different developmental modes (synarthromeric, protarthrous) will help to reveal the dynamics of these changes across this model group of early euarthropods.

## Data accessibility

All data and R scripts are provided in the electronic supplementary material [30].
